# Stretchable printed device for the simultaneous sensing of temperature and strain validated in a mouse wound healing model

**DOI:** 10.1038/s41598-022-13834-6

**Published:** 2022-06-16

**Authors:** Manoj Jose, Annelies Bronckaers, Rachith Shanivarasanthe Nithyananda Kumar, Dieter Reenaers, Thijs Vandenryt, Ronald Thoelen, Wim Deferme

**Affiliations:** 1 Institute for Materials Research (IMO-IMOMEC), Hasselt University, Wetenschapspark 1, 3590 Diepenbeek, Belgium; 2 Division IMOMEC, IMEC, Wetenschapspark 1, 3590 Diepenbeek, Belgium; 3 Biomedical Research Institute, Hasselt University, 3590 Diepenbeek, Belgium

**Keywords:** Biomedical engineering, Materials science

## Abstract

Temperature and strain are two vital parameters that play a significant role in wound diagnosis and healing. As periodic temperature measurements with a custom thermometer or strain measurements with conventional metallic gauges became less feasible for the modern competent health monitoring, individual temperature and strain measurement modalities incorporated into wearables and patches were developed. The proposed research in the article shows the development of a single sensor solution which can simultaneously measure both the above mentioned parameters. This work integrates a thermoelectric principle based temperature measurement approach into wearables, ensuring flexibility and bendability properties without affecting its thermo-generated voltage. The modified thermoelectric material helped to achieve stretchability of the sensor, thanks to its superior mechano-transduction properties. Moreover, the stretch-induced resistance changes become an additional marker for strain measurements so that both the parameters can be measured with the same sensor. Due to the independent measurement parameters (open circuit voltage and sensor resistance), the sensing model is greatly attractive for measurements without cross-sensitivity. The highly resilient temperature and strain sensor show excellent linearity, repeatability and good sensitivity. Besides, due to the compatibility of the fabrication scheme to low-temperature processing of the flexible materials and to mass volume production, printed fabrication methodologies were adopted to realize the sensor. This promises low-cost production and a disposable nature (single use) of the sensor patch. For the first time, this innovative temperature-strain dual parameter sensor concept has been tested on mice wounds in vivo. The preliminary experiments on mice wounds offer prospects for developing smart, i.e. sensorized, wound dressings for clinical applications.

## Introduction

The Greek physician Hippocrates wrote in BC 400, “In whatever part of the body excess of heat or cold is felt, the disease is there to be discovered”^1^. Recent medical literature indicates that local body temperature variations of a few degrees Celsius arise as a response to diseases or a stimulant^2,3^. Paying closer attention to such variations would enable early detection of bio-physical changes or malfunctioning of a particular part of the body. Breast cancer diagnosis and pressure ulcer detection based on monitoring the local temperature changes are some of these examples^4–6^. In a similar way to body temperature, it is pertinent to track human breath movements, real-time motion and muscle contractions to perform comprehensive health monitoring, especially for elderly people, children and athletes. Human–computer interaction, soft robotics and artificial skin research also emphasize the necessity of strain measurement and control. There are instances where advanced wearable platforms demand both temperature and strain to be measured for the same part of the human body. Wound monitoring is an example case where temperature and strain are vital parameters to be monitored.

Wound temperature and healing stages are interrelated and temperature is a significant biomarker associated with impaired wound healing. A rise in temperature at the wound site may be symptomatic of infections, inflammation or hyperemia. The temperature of the wound displays abrupt changes once it gets infected. Such a rise in temperature at the wound spot is an early predictor of chronicity of wounds even before visual changes happen. Studies of Horzic et al. shown a rise in temperature for first three days after the wound formation, which is an indication of the inflammatory period. The persistence of increased temperature even after three days is considered to be an infected and disturbed wound^7^. Contrary to it, the wound temperature decrease with respect to the rest of the body might be an indicator of low collagen deposition, reduced late-phase ‘regenerative’ inflammatory cells and fibroblasts^8^. Literature reports a 1 to 4 °C temperature difference between the normal skin and an infected wound, which is significant in detection and diagnosis^3,9,10^.

Previous studies also imply that the right amount of tensile stress on the wound site encourages neovascularization and cell proliferation. The mechano-transduction process enhances the proliferation and migration of fibroblasts and collagen synthesis. In vivo and in vitro studies show that mechanical tension is a vital requirement for the fibroblast to myofibroblast differentiation^11,12^. Based on this observation, sub-bandage pressure therapy is common in wound treatment, which demands a certain defined pressure (35 mmHg to 45 mmHg) on the wound dressing^13,14^. On the contrary, too much pressure can cause tearing the new tissue from the wound healing site. Researchers already identified the relevance of tensile forces on the wound for better healing, however, they lack feasible gauge methods. To monitor such parameters of the wound, the dressing must be transformed into a smart wearable sensing platform. Although the commercially available temperature and strain sensors are pretty accurate, the wearability is a primary downside due to its 3D shape, limited stretchability and conformability. Modern research in wearable electronics is looking at the prospect of overcoming such limitations^15^.

A crucial advancement of flexible electronics and wearable computing is witnessed in the last few decades, addressing some key issues and revolutionizing robotics and the personal healthcare sector. Wearable sensor based devices ranging from the activity tracker wrist band, to the breast cancer detecting bra and even wearable emotion detectors are developed over the last few years^16–18^. Temperature, strain, cardiac signals, moisture and motion are the common parameters monitored in a wearable platform. The multiple sensor integrated patches are highly promising for e-health care to monitor several biomarkers and it is stipulated for comprehensive personal health assessment. A recently published work presents a multisensory array for artificial skin which can sense multiple stimulants like temperature, touch and pain^19^. Yoon et al. developed a multi-layer sensory patch working in response to emotions and perception of skin temperature and conductivity^18^. Despite the advanced innovations, the human body motion artifacts and mechanical mismatch between the skin and sensors are the biggest challenges in wearable sensor precision^20^. Most of the above works employ well-accepted cleanroom-based micro-nano processing techniques for sensor fabrication that mostly consume conventional and emerging electronic materials. However, these processes are typically expensive, complex and time-consuming and the developed sensors often do not possess the required wearable features.

The introduction of printed electronics into flexible electronics fetched even more intriguing wearable applications. Printing is an alternative approach for conventional microfabrication methods that have the advantage of simple, low cost and high throughput production of sensors. In contrast to the microfabrication techniques, printing and coating techniques have an edge in creating conformable devices for wearable applications as the process is compatible with flexible and stretchable substrates and the inks being low temperature curing materials^21,22^. Printed flexible temperature sensors based on the temperature coefficient of resistance of PEDOT:PSS are described and they assert it as insensitive to humidity^23^. An alternative study reports about a thermocouple junction formed with silver and carbon, printed on PET foil and the device is flexible and bendable^24^. Similarly, towards prosthesis applications, researchers demonstrated a printed strain sensor on foil with PEDOT:PSS as the functional element^25^. A flexible screen printed strain sensor made up of conductive-resistive composite materials was developed for wearables and it has shown high sensitivity^26^. The temperature and strain sensors mentioned were mostly flexible, nevertheless, the stretchability of the sensor was the major downside.

However, the recent developments in material and device engineering unveiled plenty of prospects in stretchable wearable electronics. Recently reported carbon nanotube and silver nanowire-based strain sensors are excellent for stretchable applications^27,28^. Thermoelectric principle-based strain sensors fabricated with electro spun PEDOT:PSS together with Lycra showed a new type of wearable, stretchable strain sensor^29^. Zhang et al. have developed a micro-frame supported device with a stack type layer architecture that can simultaneously measure the pressure and temperature towards artificial skin development. The authors use the thermoelectric and piezo-resistive properties of the material for temperature and pressure measurements, respectively^30^. A dual-mode sensor has been designed with microporous polypyrole-coated graphene foam, which measures pressure and temperature with the changes in current and voltage, respectively, without a cross interference^31^. Yin et. al also worked in the similar lines and developed a flexible pressure–temperature dual parameter sensor applying a polyurethane/carbon nanofiber sponge as a sensing element^32^. This sensor has been integrated inside the mask to monitor the real-time human respiration and has been applied in a robotic arm for high temperature alarming and automatic evacuation. The development of a skin-like stretchable array of multi-functional (MF) sensors including temperature, pressure and gas sensors has been reported in literature. This sensor utilises a single sensing material of polyurethane foam coated with multi-walled carbon nanotube/polyaniline nanocomposite for all the three sensor mechanisms^33^. In another work, a stretchable and highly durable temperature sensor on textiles was developed, though the stretching induced a significant shift in the temperature measurement characteristics^34^.

As per the literature, temperature-strain sensing has rarely been explored for wound monitoring applications. Authors of this article for the first time according to their knowledge, present in this study, a temperature-strain dual parameter simultaneous sensing methodology exclusively targeting wound monitoring applications. Initial research attempts in this area show commercially available non-conformable sensors integrated into a wound patch^35,36^. Researchers from Nanyang technological university demonstrate 3D aerosol jet printed silver patterns for strain sensing for bandages. Escobedo et al. presented individual temperature and strain sensors integrated into a polyamide foil patch for wounds, tested on a human body analogue. However, they did not show the cross-sensitivity of each stimulus to the other^37^. In a significant further development, resistance monitoring of temperature and strain via a sensitive hydrogel towards wound monitoring was described, however, both parameters interfere with each other due to same readout parameter^38^. Moreover, the hydrogel was tested on mice for antimicrobial properties though the sensor has not applied for temperature and strain measurements. There are considerable efforts to prepare conformable, stretch-insensitive temperature sensors to make precise temperature monitoring for efficient wound monitoring. Likewise, researchers identified and are putting efforts to realize highly stretchable strain sensors to regulate the tensile forces on a wound, in turn, optimise the wound healing conditions.

The smart wound dressing is a multivariate concept and needs to meet many rigorous wearability, functionality, compatibility and disposability prerequisites:

- Highly stretchable, flexible sensors with a soft interface to the skin
- Comprehensive wound monitoring through multiparameter evaluation
- Higher sensor integration density and they preferred to be multifunctional
- Sensors need to possess high accuracy, sensitivity and consistency
- Ease of fabrication & material availability
- Disposability and cost-effectiveness
- Transparency and biocompatibility

This work assiduously develops a flexible and stretchable dual parameter thermoelectric sensor for wound monitoring in vivo*.* This innovative single sensor concept has two different readout parameters, i.e. temperature and strain, designed to not interfere with each other during simultaneous measurements. The envisaged sensor uses printing techniques that ensure the industry compatible and facile fabrication process on wearable substrates and cost-effectiveness. The projected dual parameter sensing device could improve the efficacy of wearable sensor applications by simplifying the sensor system and the readout instrumentation. Competent personal health care demands precise, flexible and wearable sensors to sensing. This work uses a soft interfaced and highly stretchable thermoplastic substrate and an inhouse formulated PEDOT:PSS ink for the sensor fabrication. The smart selection of PEDOT:PSS has been utilized as a thermoelectric material due to its low toxicity, ubiquity, elasticity and ease of processing. The PEDOT:PSS material ensures the sensor's transparency, which helps the wound to be visually monitored without removing the bandage. The sensor effectivity is tested in real life scenarios and the second part of the manuscript emphasizes this via in vivo tests on mouse wounds. These experiments show the applicability of this dual parameter and wearable sensor for real-life scenarios.

## Methods and materials

**Figure 1:**
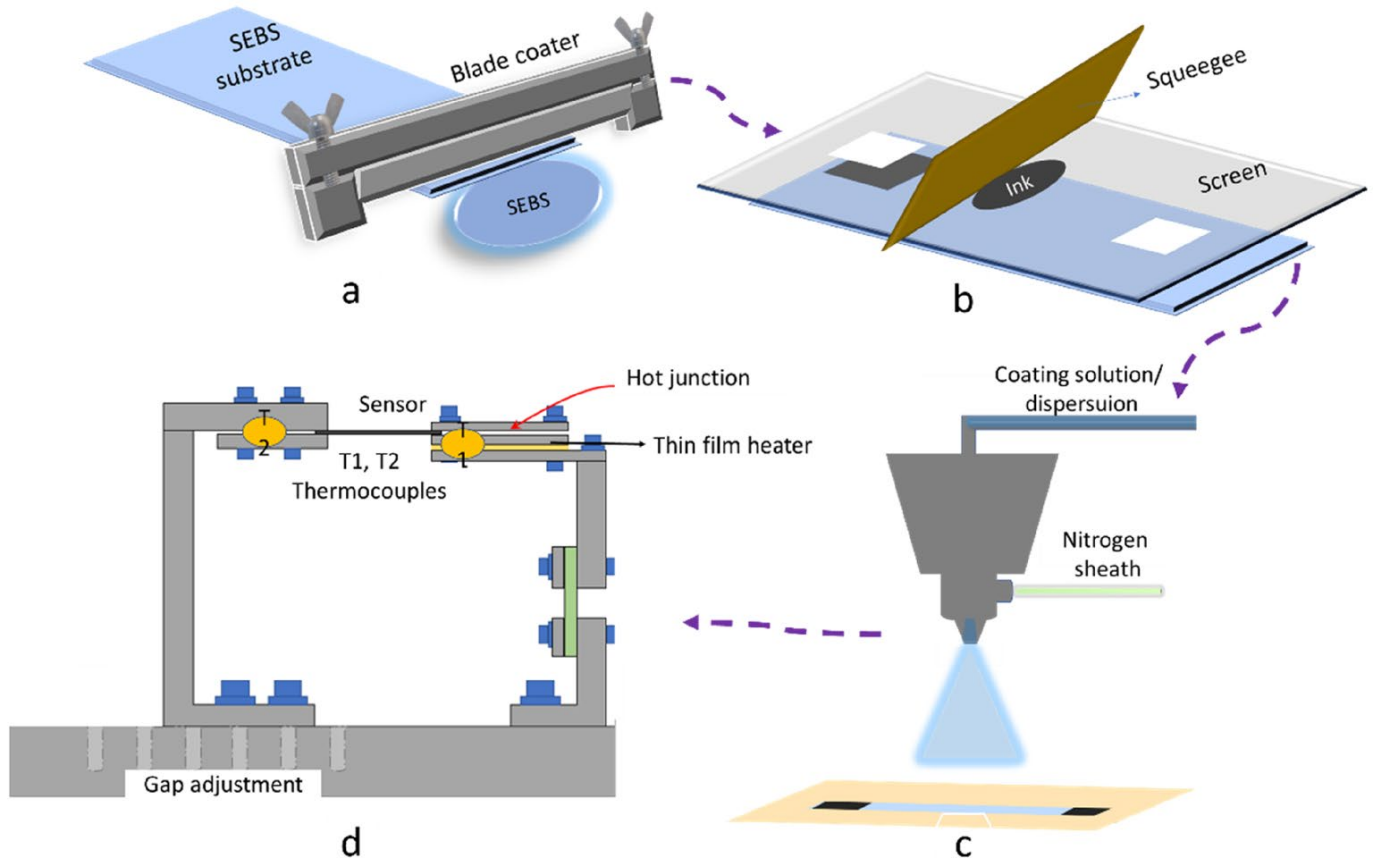
SEBS substrate preparation with blade coating (**a**); Screen printing silver electrodes on SEBS (**b**); Spray coating PEDOT:PSS based composition on SEBS substrate which is selectively masked (**c**); Temperature sensor characterisation set up (**d**).

This work uses styrene ethylene butylene styrene (SEBS) polymer pellets supplied from Asahi Kasai corporation, Tokyo, Japan for the substrate preparation. The SEBS pellets were dissolved in chloroform at a ratio 1 g/3 ml and were blade coated onto a glass plate (Fig. 1a). The coated SEBS layer on the glass plate was cured in ambient air drying and then removed it as a foil (approx. thickness 1 mm) from glass plate^18^. Previous studies prepared the foil with the same material and demonstrated excellent elastic properties of up to 800% stretchability^39^. (The hysteresis behaviour of the substrate stretching can be found in the supplementary info, Fig. S1). The sensor consisted out of two silver electrodes connected by a stretchable PEDOT:PSS layer. Here the silver electrodes are 6 cm apart and they are screen printed onto the SEBS substrate (Fig. 1b). Flat-bed screen printing (ISIMAT 1000 PE, Ellwangen, Germany) with a 40 × 40 meshed PET screen (SEFAR PET 140/355–31, 40 threads per cm) is used here. Stretchable silver paste PE873 from Dupont (60% solid content, curable at 110 °C, 20 min) was used for realizing the silver electrodes of 1 cm X 1 cm. A conductive polymer PEDOT:PSS in the brand name PH1000 from Heraeus, Precious Metals GmbH Co.KG, Hanau, Germany was used in the present work. Lithium bis(trifluoromethanesulfonyl)imide (LiTFSI), 99.9% extra-dry salt was purchased from Solvionic SA, Toulouse, France used here as a stretchability enhancer. LiTFSI salt was added to the PEDOT:PSS dispersion with different weight ratios and vigorously stirred and mixed with the help of a speedmixer (Hauschild model DAC 600.2 VAC-P). To make the composition spraycoatable, the formulation was diluted with water in the ratio of 1:4.

The mentioned PEDOT:PSS-LiTFSI composition has been deposited with the help of an ultrasonic spray coater (USSC) (Fig. 1c). The deposition was completed with 20 layers with flow rate of 0.15 ml/ min. Here the thin film deposition technique USSC was selected, as it is a versatile, non-contact, large-area solution/dispersion coating technique. The ultrasonic vibration of the coating head inducing standing waves on the solution drop leads to its atomization, provides consistent drop size, and a high degree of coating uniformity on flexible and rigid substrates. The superior quality of the PEDOT:PSS layer ensures better transparency and electrical properties of the printed thin film. USSC has found great importance in high precision thickness (from nanometre scale) and uniform active layer deposition of solar cells, light-emitting diodes, sensors, and other functional coating applications, specifically in mass volumes^40–42^. The prepared samples were then treated with aqueous solutions (1 wt%) of sodium thiosulphate (Na_2_S_2_O_3_) for 30 s and then rinsed with ethanol. Finally, it was dried with a nitrogen gun.

The sensor fabrication for wound investigation on mice was carried out on commercial thermoplastic polyurethane (TPU) transparent foils (TPU is frequently applied in wound bandages and SEBS needs further studies to use for such application). This stretchable TPU foil was received from Grafytip, Houthalen, Belgium. Silver electrodes have been screen printed and the PH1000 PEDOT:PSS formulation diluted with water in the ratio of 1:4 is coated on the TPU foil using the USSC method.

### Measurement setup

The sensor temperature and strain measurements are carried out in two different set ups. The temperature sensor characterization is conducted inside an oven chamber where the temperature and humidity can thoroughly be controlled. Based on the work of Alhazmi et al. on a thermal conductivity measurement setup^43^, a characterisation system is in-house built and slightly modified for the temperature sensor characterization (Fig. 1d). The measurement setup consists of a cold and hot arm where a sample can be locked in-between. The hot arm has a thin film heater integrated, combined with a highly thermal conductive graphite sheet to evenly spread the heat distribution on the pile bed. The thin film heater, rated at a maximum power of 2.5 W, can be controlled to attain a predefined hot arm temperature. One Electrode (Junction) of the sensor was locked on this hot arm. The second electrode (Junction) was locked in the cold arm, being similar to the hot arm, except from the thin film heater. Both arms are precisely coupled with thermocouples for the temperature measurement in the respective junctions. Both the electrodes are externally connected for thermoelectric voltage measurement. An Agilent 34001A multimeter is used for recording the readings. The gap between the sample holders are increased to perform stretchability of temperature sensor characterization. Strain measurements are carried out with an in-house made stretch bench where the system is controlled through a LabVIEW interface^44^. The speed of the stretching during the strain measurement was 1 cm/minute and the sensing part of the sensor consisted of 5 cm length.

The Fermi level measurements were done by Frequency modulated Kelvin probe force microscopy (KPFM) from Park NX10 with a vertical resolution 0.015 nm and lateral resolution of 0.05 nm, Pt/Ir coated tips from ST instruments were used to perform the experiment. All the experiments were conducted in the ambient condition. Before the measurements, the conductive tips are calibrated.

### Principle of working

**Figure 2:**
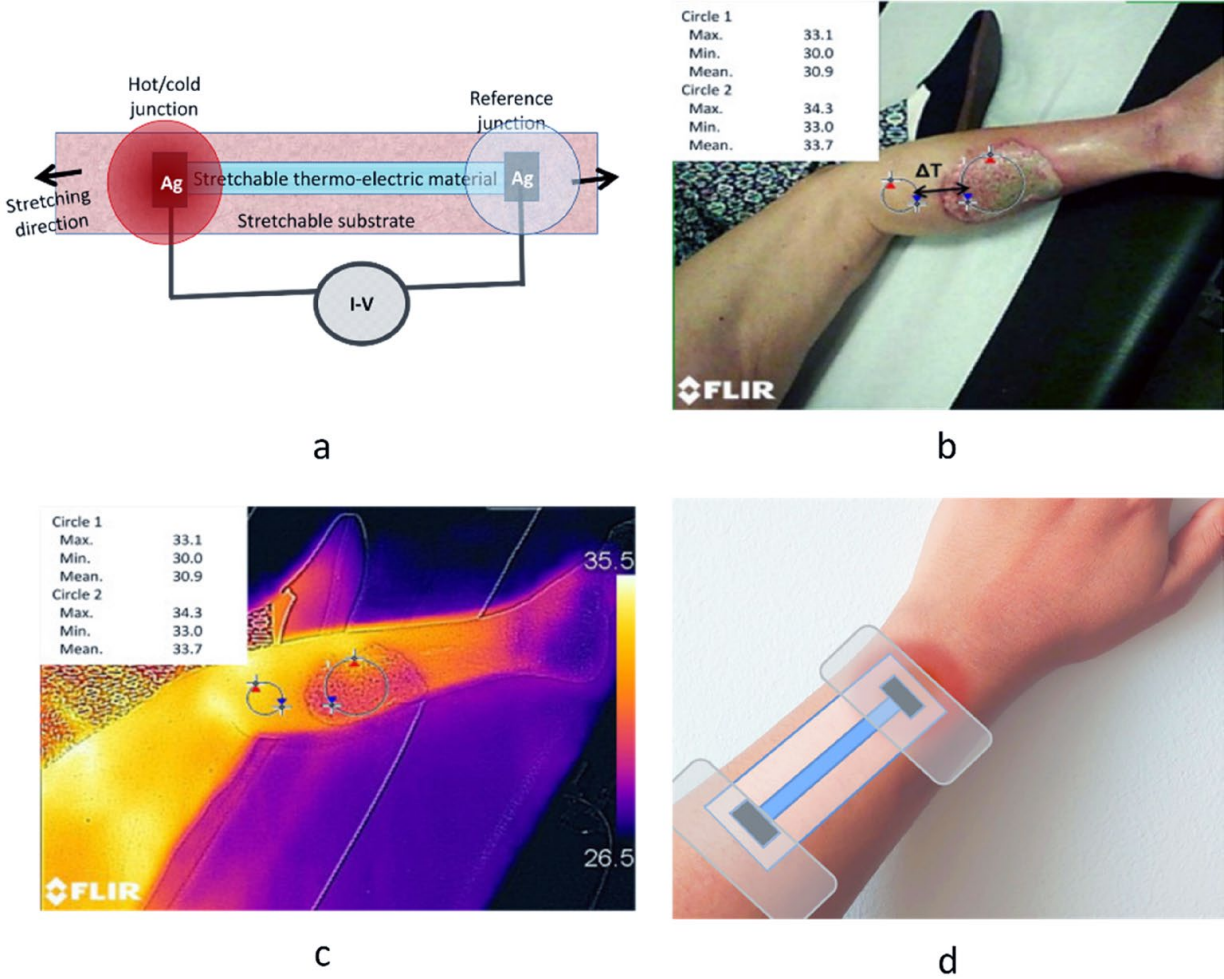
Illustration of the strain and temperature sensing in a single device which has the build-up of a stretchable substrate which is printed with a thermoelectric material and the junctions are formed with printed silver (Ag) electrodes (**a**);; Photograph of the wound (**b**) and its FLIR thermal image where the temperature difference between the wound and surrounding tissue is visible (**c**) ( Image **b** & **c**^46^, are reproduced with permission from Elsevier (License number 5111310168119)). Schematic of the sensor attached on a wound site (**d**).

In the 1800s, a German physicist, Thomas Johann Seebeck discovered that two dissimilar metal junctions kept at different temperatures generate an electromotive force (emf), later named as Seebeck effect. The Seebeck effect is a peculiar type of a thermodynamic phenomenon, where fluxes of electrons and heat energies are mediated, leading to a coupling between the electrochemical potential and the temperature*.* Here, the flow of electrons from the hot junction to the cold junction generates an emf, which is proportional to the temperature difference between both junctions. The ratio of this induced thermoelectric voltage to the temperature difference is called the Seebeck coefficient which is an intrinsic property of the material^45^. Our search for wearability and fabrication attributes such as printability, flexibility and biocompatibility along with reasonably good thermoelectric properties, leads to conducting and semiconducting polymers. Thus, the choices of materials like Polyaniline, P3HT and PEDOT:PSS are evaluated based on their properties. The superior mechano-electrical transduction properties of PEDOT:PSS helps the sensor to be flexible and stretchable. The stretching-induced strain can be indicated based on the resistance variations due to the dimensional changes of the PEDOT:PSS layer. The architecture of the proposed device is simply a thermocouple and the thermoelectric element between the junctions acts as a stretchable conductor. Figure 2a shows the device design consisting of a flexible substrate on which planar structures are printed so that it can act as a temperature and a strain sensor at the same time. The stretching of the thermo-electric layer proportionally induces a resistance change on the device. However, it has no significant influence on the differential temperature measurement since the generated voltage is measured at open circuit conditions^30^.

The temperature measurement of the proposed sensor is novel and different from the conventional thermocouple systems. In standard thermocouple measurements, one of the thermocouple junction is on the object/point of the intended temperature measurement. Furthermore, the second junction is the reference junction, where its temperature is known. The generated voltage is proportional to the temperature difference (ΔT). The unknown temperature at the first junction, can calculate from the known Seebeck coefficient of the material and the reference junction temperature. Studies in the past shown the ΔT between the wound and its neighbouring healthy body area as a pointer of infection and inflammation (Fig. 2b,c), where the temperature of the wound and its adjacent area is mapped with thermal imaging^46,47^. However continuous monitoring of wounds with a thermal camera inside a wound dressing is nearly impossible and such devices would be expensive. In the proposed measurement scheme of this work, one junction is set to be on the concerned area of the skin (wound) to be monitored. The conjecturing healthy skin, a few centimetres away from the wound, is taken as a reference junction, where the ΔT between the wound and normal skin generates a thermoelectric voltage (Fig. 2d). This is a relative temperature measurement where the healthy tissue becomes a reference of the measurement. This eliminates the necessity for a temperature compensated 2nd junction which is a stipulated requirement in thermocouples. The innovative design concept for wound temperature monitoring circumvent complexities that would stay on the conventional thermocouples. This is not a universal solution to all temperature and strain measurements, but it stays relevant and remains as an innovative solution to wound temperature and to define mechanical forces at the wound site.

### Mouse wound model

To demonstrate the applicability of the developed sensor, a mouse wound healing model, i.e. ‘full thickness excisional wound model’^48^ was applied. All experimental animal procedures were approved by the Ethical Committee for Animal Experimentation (ECAE) UHasselt (approval number 201946) following the guidelines described in Directive 2010/63/EU as well as the specific Belgian law (Belgian law of animal welfare and Royal Decree of 29 May 2013) on the protection of animals used for scientific purposes. In addition, this pilot feasibility study is reported in accordance with ARRIVE guidelines . Mice were housed in a temperature-controlled room (20 ± 3 °C) on a 12 h light–dark schedule and with food and water ad libitum. Wounds were introduced in male BALB/cJRj mice of 11 weeks old (Janvier, Le Genest-Saint-Isle, France). Mice were anesthetized with 2% isoflurane (IsofFlo, Abbot Animal Health, Belgium) during all surgical procedures and wound measurements. First, dorsal hair was shaved, followed by a depilatory cream (Veet, Reckitt Benckiser Group, Slough, UK) to remove the remaining hair. The next day, the dorsum was disinfected with an iodine solution and full thickness round wounds just below the nape of the neck were made using a biopsy punch (Stiefel) with a diameter of 10 mm which punctures the epidermis. The *panniculus carnosus* was removed using curved Vannas spring scissors (Fine Science Tools, Heidelberg, Germany). The wound was covered a semi-occlusive dressing (Tegaderm, 3 M, Diegem, Belgium), impermeable to liquids, bacteria, and viruses.

Finally, mice were placed in individual cages, and allowed to fully recover from anaesthesia on a heating pad. At several time points (4th, 6th, 8th and 12th of days post-surgery), mice were again anaesthetised, the tegaderm was removed and the wound temperature was measured using the developed sensor. Every time the sensors were attached on the wound and removed after the measurements. The sensor was placed in such a way that one electrode junction was on the wound and the second junction was at the top of the cauda line region of the mice. The foil sensor has a liner sheet which is removed first and the sensor has been attached to one junction. On the backside of the substrate, where the silver electrode is printed, support was foreseen with a non-stretchable tape, so that the electrode junction does not get damaged under extreme sensor stretching conditions. Then it is stretched to the required pre-defined length and the second junction is attached in such a way that it connects the wound position and the reference spot. The junctions were firmly placed on its respective position with the help of a medical tape.

## Results and discussion

### Strain sensing

**Figure 3:**
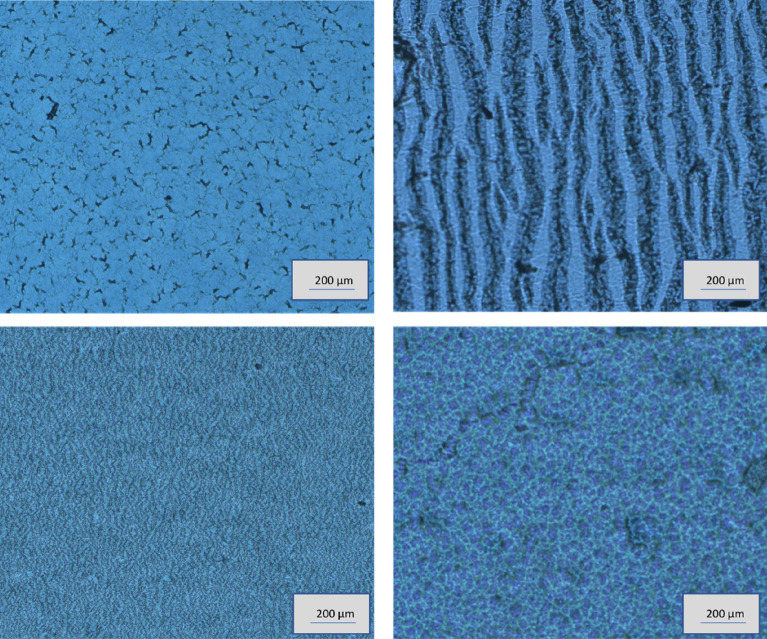
Microscopic images of PEDOT:PSS layers with varying proportions of stretching enhancers kept for stretching. Layer morphology of PEDOT:PSS composition with no addition of stretchability enhancers (**a**), after stretching it to 100% strain value (**b**), the resultant layer of PEDOT:PSS with stretchability enhancer of 5wt% and 15wt% respectively at 100% strain condition (**c**, **d**).

Wearable strain sensors have seen a massive rise in demand due to their role in translating human organ functioning, wound monitoring, and body movement detection into electronic information. Conventional strain sensors are less ideal for health monitoring applications mainly due to their rigidity and stiffness that rather than making the sensing complicated, restricts free body movements. Wearable strain sensors are expected to be essentially highly stretchable, soft interfaced and conformable to accommodate multiscale and dynamic deformations caused by body movements. In this article, regarding the strain sensor development, the authors primarily focused on improving the stretchability of the PEDOT:PSS layer as the previously reported PEDOT:PSS based sensors were inferior in stretchability. Enhanced stretchability of the sensor offers better transduction of the body movements into sensor signals, but demands a lower Young’s modulus value of the material. Wang et. al published a new approach to enhance the elastic properties of PEDOT:PSS material with the addition of stretchability enhancers^49^. In this work, we deployed LiTFSI salt as the stretchability enhancer in the preparation of the ink formulation. Three different ink formulations were prepared with compositional differences; PEDOT:PSS with no additives, PEDOT:PSS with 5wt% enhancer and PEDOT:PSS with 15wt% enhancer. The USSC coated samples on the SEBS substrate underwent chemical treatments to improve the temperature sensitivity (explained in detail in the section discussing about the temperature sensor). The PEDOT:PSS-LiTFSI layer on foil was stretched (100%) and stuck to a glass slide using adhesive tape and it was visualized with the help of an optical microscope (Fig. 3). The first sample prepared without the enhancer additive showed relatively large breakages in the layer surface. The samples with 5wt% enhancer showed better resilience to layer breakages than the former one. However, the sample with 15wt% enhancer exhibited an exceptionally high integrity and much less defects and rips. The addition of the stretchability enhancer to the PEDOT:PSS composition leads to the charge screening effect, reducing the Coulomb attraction between PEDOT and PSS^49^. The PEDOT phase modifies itself into an interconnected nanofibrous and a crystalline morphology. The stretchability enhancer stays in the more amorphous PSS domains, providing a softer matrix for PEDOT^50^. These structural changes help to achieve the reversible stretchability of the strain sensor and resilience to the formation of cracks and damages in the sensing layer. Samples prepared with 15wt% LiTFSI salt are chosen for further investigations in this work.

The resistance (R) of the thin and conductive layer of PEDOT:PSS having a length l, a width w, and a thickness (t) is given as:

1 $${\text{R}} = { }\frac{\rho l}{{wt}}$$

Stretching persuaded strain (ε) leads to a corresponding increase in length, meanwhile, width and thickness decreases proportional to Poissons ratio of the PEDOT:PSS (υ_p_) and the substrate (υ_s_). This is based on the assumption that a resistance change is occuring from the stretchable conductor's deformation and the resistivity (ρ) of the PEDOT:PSS material is not changing during stretching. The new resistance R' after stretching is given by

2 $$R^{\prime} = \frac{{\rho l\left( {1 + \varepsilon } \right)}}{{w\left( {1 - v_{s} \varepsilon } \right)t\left( {1 - v_{P} \varepsilon } \right)}}$$

**Figure 4:**
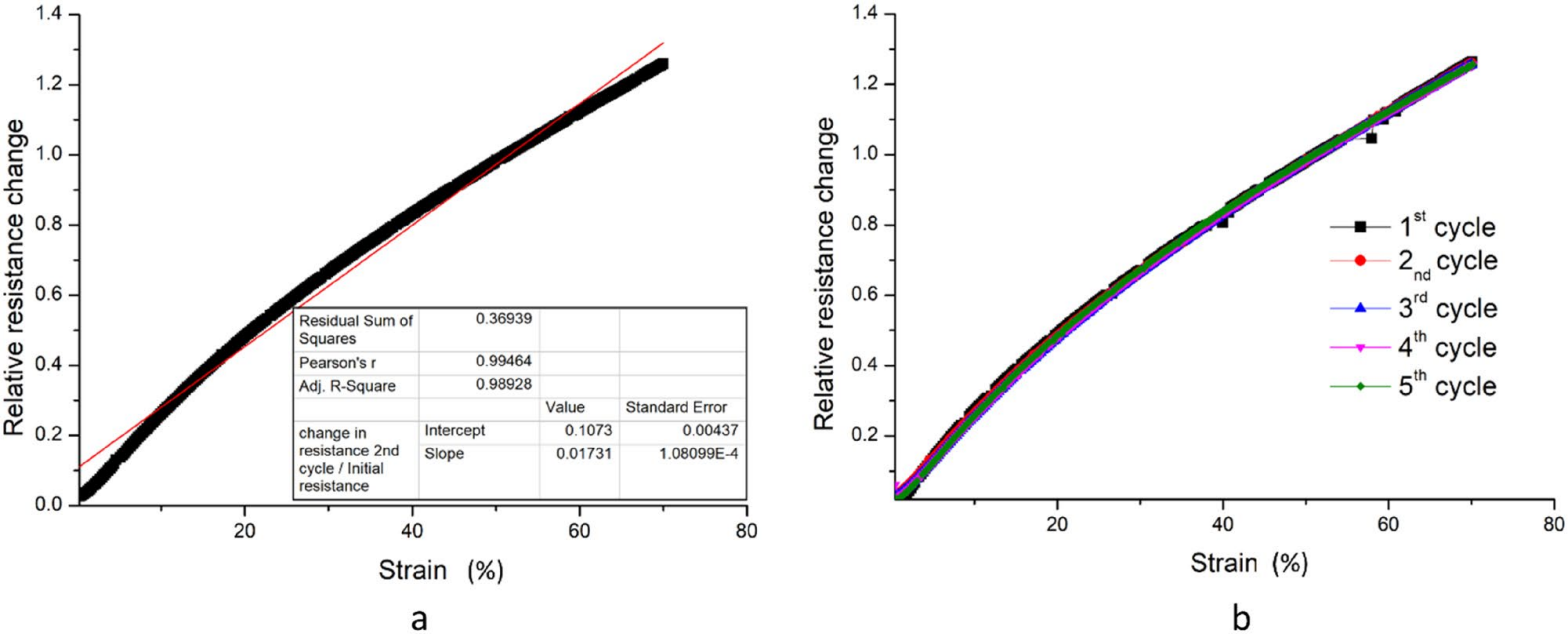
Normalised resistance change of the sensor for 70% strain (**a**) and demonstration of reproducibility of the sensor measurements (**b**).

The developed strain sensors are characterised and tested for their efficacy based on the scales of measurable range, linearity, repeatability, consistency and the geometric factor. The proposed sensor (unstretched sensor resistance of 1840Ω ) exhibits excellent stretchability on an input scale of 0 to 70% strain (Fig. 4a). The characteristic curve of strain to relative resistance change shows a linear transfer function of the sensor with a correlation coefficient of ∼ 0.99. The sensor was tested repeatedly with an applied strain value corresponding to 70% (Fig. 4b) demonstrated a relative resistance change of 1.26 ± 0.002. The minor standard error indicates the capability of the sensor to transduce sensor signals repeatedly identical (Sensor measurements with the standard deviations have been included in the supplementary info, Fig. S2).

**Figure 5:**
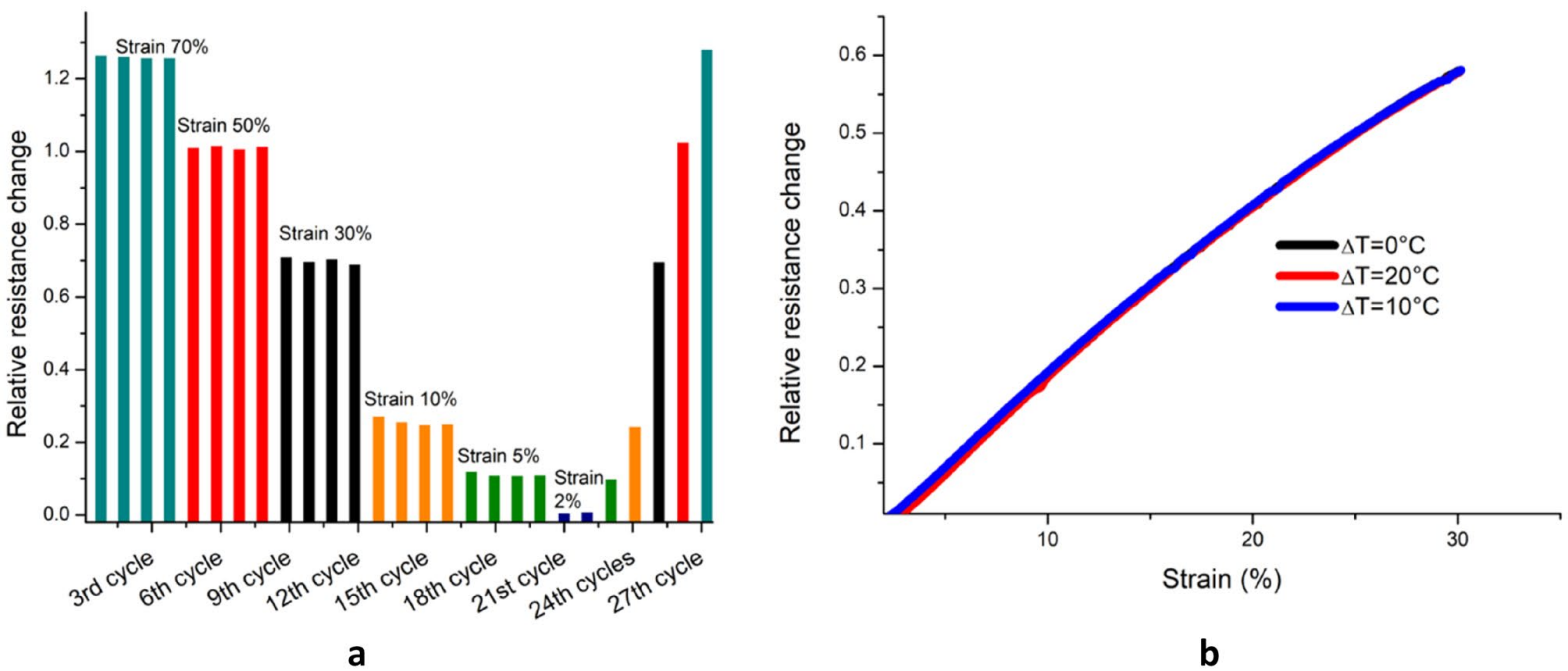
Plot between applied strain from 2 to 70% and corresponding induced resistance change for repeated cycles (**a**), strain sensing for different ΔT set across the junctions to ensure the null cross over sensitivity (**b**).

The geometric factor (GF) is a measure of the sensitivity for the strain sensor and a higher GF indicates better sensitivity. It is measured as the ratio of the relative resistance changes to the applied strain values. The developed sensor demonstrates a GF of 2 up to 50% strain values and 1.8 up to 70% . This is a fairly good value of GF in comparison to those of the previously reported PEDOT:PSS based strain sensors^29,51,52^. Several works in the literature reported the enhancement of strain sensor GF through specific strategies like a plasma treatment of the substrate, a high functional layer thickness, and defect formation via cracking^53^. Such sensors however are demonstrated to be inferior in other attributes such as linearity and repeatability^27,54–56^. This work emphasises the intrinsic stretchability of the functional layer, through which the sensor attributes are improved for strain sensing. The resistance changes corresponding to the ascending strain cycles with strain values ranging from 2 to 70% confirms the repeatability and precision in the measurements (Fig. 5a). This is attributed to the reversible deformation of the homogenous matrix of stretchable PEDOT:PSS-LiTFSI salt composite layer. The changes in resistance for the descending strain cycles with strain values ranging from 70 to 2% confirms the consistency in the measurements in reference to the ascending cycles. Additionally, the strain sensing has been investigated for cross-talk of the differential temperature at the junctions. Figure 5b reveals there is no consequential impact on the strain measurements from any of the three differential temperature set points. Precise and consistent strain measurement over a wide range with excellent sensitivity makes this sensor suitable for wearable applications.

### Temperature sensor

As a response to the biophysical changes of the wound, the human body generates local temperature variations that either indicate infection or act as a biomarker for wound diagnosis. Fierheller and Sibbald explored the relation between peri-wound temperature and infection in chronic wounds of 40 patients using a handheld infrared thermometer. They measured that infection causes an average of 2.46 °C temperature rise from the reference body temperature^57^. To draw such real-time vital information regarding the wounds without removing the wound dressing, one would need temperature sensors with mechanical properties similar to that of the skin to perform non-invasive accurate temperature sensing.

The temperature sensor investigated and developed in this work has been tested and characterised in conditions as specified in the ‘measurement set up’ section. One of the junctions of the sensor is kept at a controlled rising temperature (heater coil) and the second is maintained at the thermal oven temperature. The differential temperature set across two junctions generates a thermo-electric voltage which is in direct proportion to the temperature. Thus, this measured voltage is the product of the temperature difference (ΔT) between the two junctions and the Seebeck coefficient of the thermoelectric material. Assuming that the temperature sensor sensitivity solely relies on the Seebeck coefficient of the materials, this property has considerable importance in enhancing the sensitivity of the temperature measurements^58,59^**.** Previous works studied the tailoring of the electronic band structure of the PEDOT: PSS materials for enhancing their thermoelectric properties^60,61^. The electronic states formed in the band structure of PEDOT:PSS , constitute to the Density of States (DoS), which can be filled/vacated by redox processes. At very low dopant concentration, the DoS of PEDOT:PSS behaves like a conventional inorganic semiconductor and at high doping, it resembles a metal band structure. This contradictory behaviour arises from the high conductivity achieved at high doping levels, where the charge-transport energy level (E_t_) and Fermi level (E_f_) comes closer. Based on Boltzmann’s transport equation, the Seebeck coefficient (S) and the DoS are related through Eq. (3)^62,63^:

3 $${\text{S }} = \frac{{k_{b} }}{{\text{e}}}\left[ {\left( {\frac{{E_{f} - E_{t} }}{{k_{b} T}}} \right)} \right]$$

Here K_b_ is the Boltzmann constant, T the room temperature and e is the electron charge. As the charge carrier concentration decreases, the gap between the fermi energy and transport level increases. This in turn, increases the Seebeck coefficient and decreases the conductivity^64,65^. Early studies have demonstrated an enhanced Seebeck coefficient for the PEDOT:PSS material when they are treated with certain electrochemical reducing agents^66,67^. Sodium thiosulphate is a known reducing agent, causing the electrochemical reduction of PEDOT:PSS^68^. In this work we have applied the sodium thiosulphate treatment for the PEDOT:PSS-LiTFSI layer and the sensor shows a sensitivity enrichment from 12 to 25 µV/°C. As expected, the resistance of the sensor increased twofold after the sodium thiosulphate treatment which is assumed to be due to the changes in the carrier concentration in the PEDOT:PSS-LiTFSI layer. This is also substantiated with the KPFM measurements of PEDOT:PSS-LiTFSI films. The treated and untreated PEDOT:PSS-LiTFSI layer shows a fermi energy difference of ~ 0.8 eV (KPFM images are given in supplementary information, Figure S4 a & b). This modification in the fermi level through a chemical treatment contributes to the better sensitivity of the sensor.

**Figure 6:**
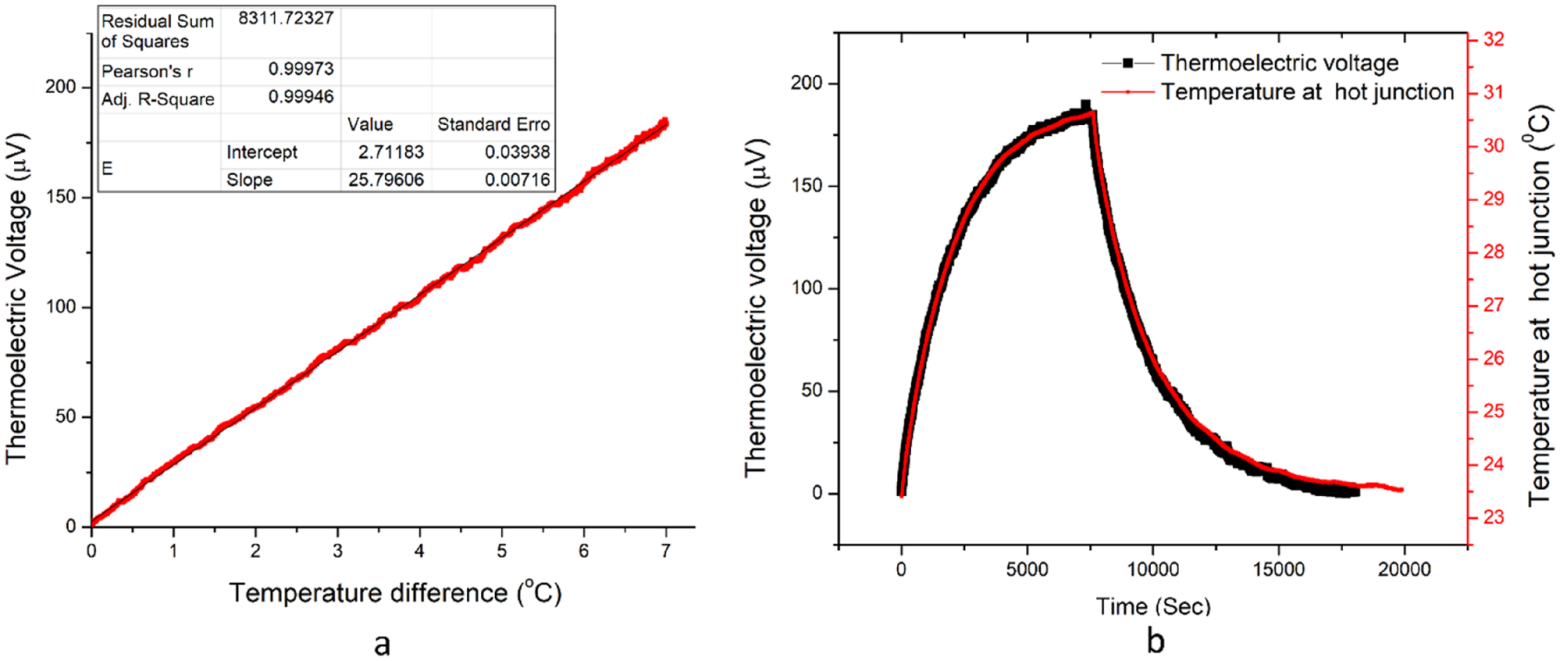
Temperature induced thermoelectric voltage in the sensor is plotted (**a**), Sensor applied for measuring the heating and cooling of heater coil shown with the proposed sensor and commercial thermocouple (**b**).

As shown in Fig. 6a, a linear relation exists between the sensor input ΔT and the voltage output with an excellent correlation coefficient of 0.999. The temperature measurements have a resolution of 0.1 °C. Figure 6b shows the temporal evolution of thermoelectric voltage generated as the sensor response to the hot junction heating. Likewise, the sensor performance is compared in reference to a commercial thermocouple where the measurements are observed to exactly coincide with each other (A plot of the same including the corresponding errors is given in the supplementary info, fig. S3). The sensor characteristics in Fig. 6b are well aligned to the real-time temperature curve and do not exhibit any hysteresis.

**Figure 7:**
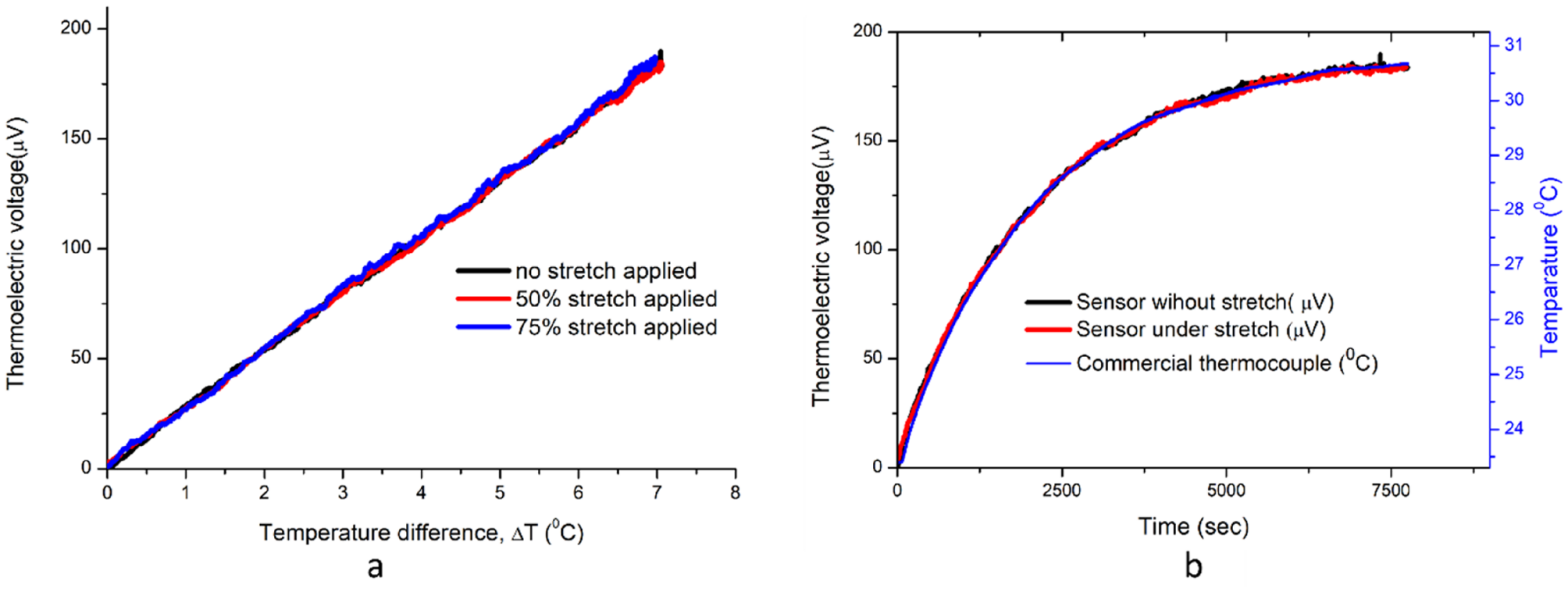
The temperature sensing using proposed sensor at different strain conditions (**a**) Temperature rise at the hot junction over time monitored with the sensor in unstretched and stretched (50% strain) conditions. Simultaneously, the hot junction temperature measurement with a commercial thermocouple (**b**).

Figure 7a shows the generated thermoelectric voltage at different sensor stretching levels against the temperature difference that exists between the junctions of the sensor. The sensor was tested for different stretching levels of 0%, 50%, and 75% by adjusting the sample holder gap in the measurement setup. The variation in the level of strain applied on the sensor doesn’t show any significant impact on the induced voltage. The measurement setup itself had a tolerance of ± 0.1 °C which could produce a negligible influence on the accuracy of different measurement cycles. These inaccuracies in the measurement system occur from the minor heating instabilities of the oven and the precision tolerance of the reference temperature measurement with the commercial thermocouples. Apart from the minor systematic errors in the thermoelectric voltage at different stretching levels, the measurements were identical for the proposed sensor. The results in Fig. 7b show the temperature rise at the hot junctions with time, monitored with the sensor being in both the stretched and unstretched modes. This result also proves that the sensor readings are analogous in stretched and unstretched sensor conditions and strictly follow commercial thermocouple reference measurements. The KPFM measurements reassured that the Fermi level is not changed with stretching the PEDOT:PSS-LiTFSI film, as shown in the surface potential image (Figure S4 a&c) in the supplementary section. It implies that the electronic band structure of the PEDOT:PSS-LITFSI film is not modified under stretching. This enables the sensor to make precise temperature measurements under different stretching conditions.

### Simultaneous measurement of temperature and strain

**Figure 8:**
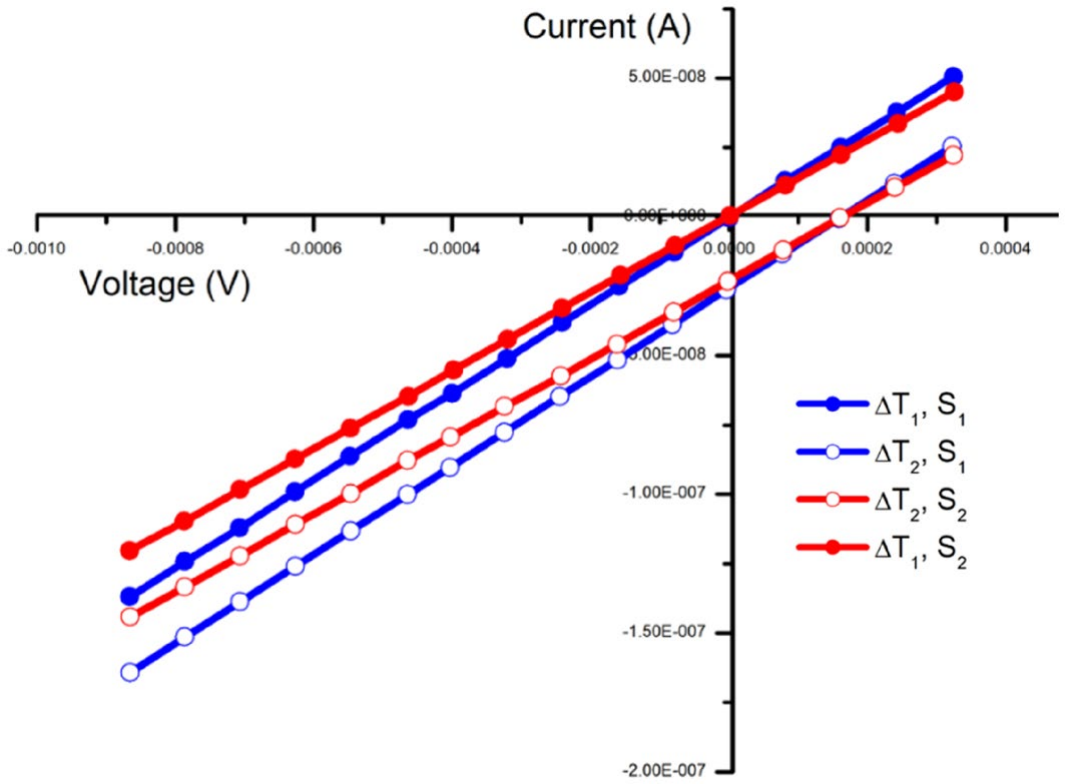
Simultaneous measurement of temperature and strain. ΔT1, ΔT2 indicates two different temperature differences and S indicates the strain. The slope of the graph gives the strain-caused resistance change of the device, while the temperature-generated voltage can be read from the voltage shift on the horizontal axis.

The concept of the dual-parameter sensor mainly addresses the challenges from the cross sensitivity of stimulants. The peculiarity of the measuring parameters, the unique design and the innovative material selection, as discussed in the previous parts, made the independent measurement of two parameters, temperature and strain, without any decoupling analysis, possible. The sensor resistance has no impact on the measured voltage at the open circuit conditions. Meanwhile, the junctions at a difference of a few degrees Celsius have negligible influence on the device’s resistance compared to resistance changes induced by the applied strain, as shown in the above section. This facilitates the concurrent measurement of these two parameters. The sensor is designed so as to sense the two parameters simultaneously with the two outputs being; the temperature induced open circuit voltage and the strain-made resistance change. This dual parameter sensing of the same device lead to the feasibility of using current–voltage (I-V) characterisation of the device. This helps reducing the measurement complexity from two independent readouts to a single I-V measurement and two sensing outputs from a single sweep. The results are shown in Fig. 8, indicating that the two parameters can be sensed independently without interfering with each other. An increase in temperature difference (from ΔT1 to ΔT2) induces an increase in open-circuit voltage. Likewise, an increase in strain (from S1 to S2) causes a resistance changes which can be seen in the change of the slope of the I-V curve. The I-V characterization of the device demonstrates the identical temperature induced V_oc_ at different strain values (open or filled dots). Meanwhile, the device shows parallel I-V plots with identical stretching conditions (red or blue curve) irrespective of the temperature difference between the two junctions.

### Sensor testing on mice wounds

**Figure 9:**
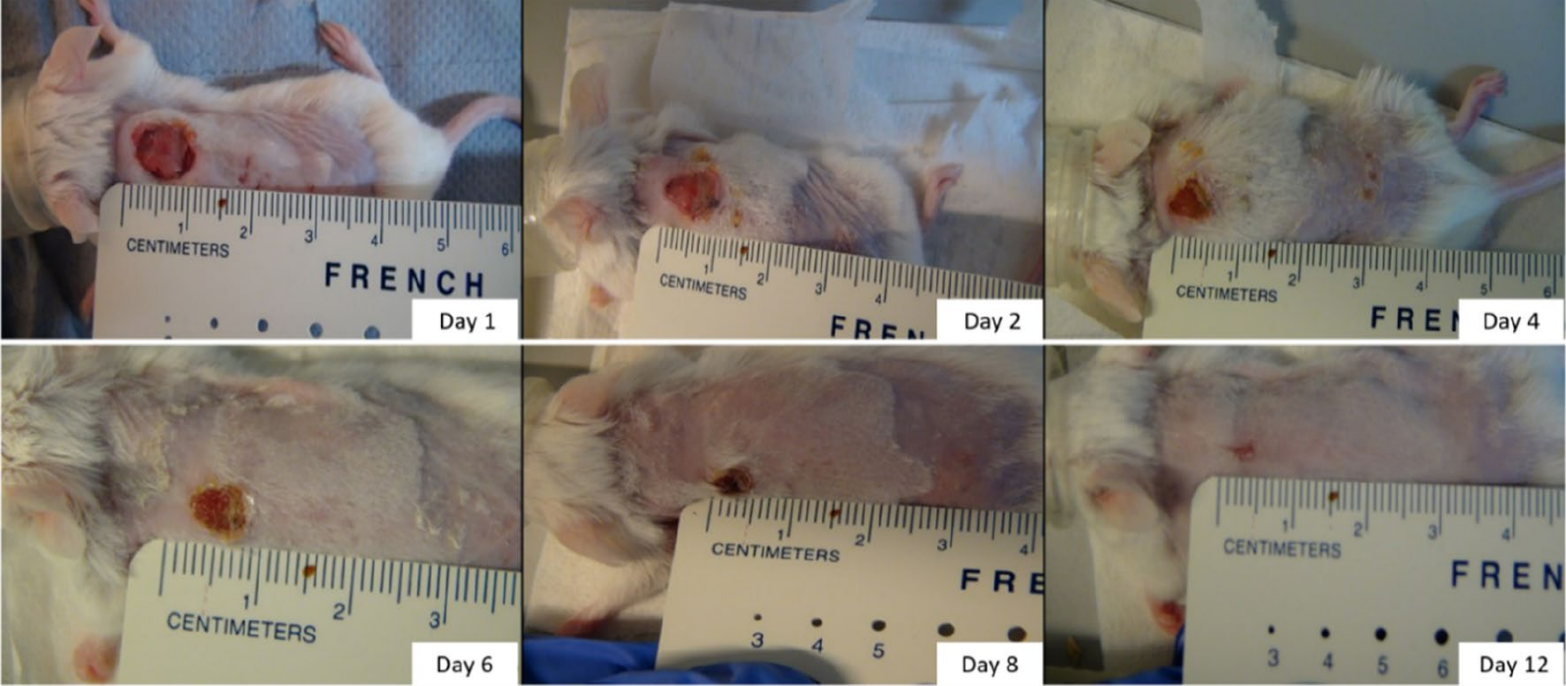
Different stages of the Mice wound closure from day 1 to day 12.

The damage in the skin epithelial integrity and the disruption of the composition, structure, and functioning of the beneath tissues are defined as a wound. In order to rebuild the wound into natural tissues, there are many complex, serial yet distinct biophysical and biochemical activities such as cell migration, proliferation, and tissue remodelling^69^. Wounds which are healing in a relatively fast and routine way are called acute wounds. However, there are many more risky and complicated wounds identified as chronic with diverse aetiology. Despite this diversity, chronic wounds are commonly diagnosed with ischemia, bacterial colonisations, and imbalance between growth promoters and inhibitors. Due to this complex and deep problematic nature of impaired wound healing, in vivo experiments on animal models are needed to understand this wound healing process. Wound studies on animals commonly opt for a mouse model as it is economical, amenable to genetic manipulation and easy to incorporate them for in-house experiments. To demonstrate the application of the proposed sensor in wounds, a mouse model was used. As described above, a 10 mm surgical wound was made at the nape of the neck of the mice. From time to time, the mice were anaesthetised while making the sensor measurements., The wound closure over time is demonstrated in Fig. 9.

**Figure 10:**
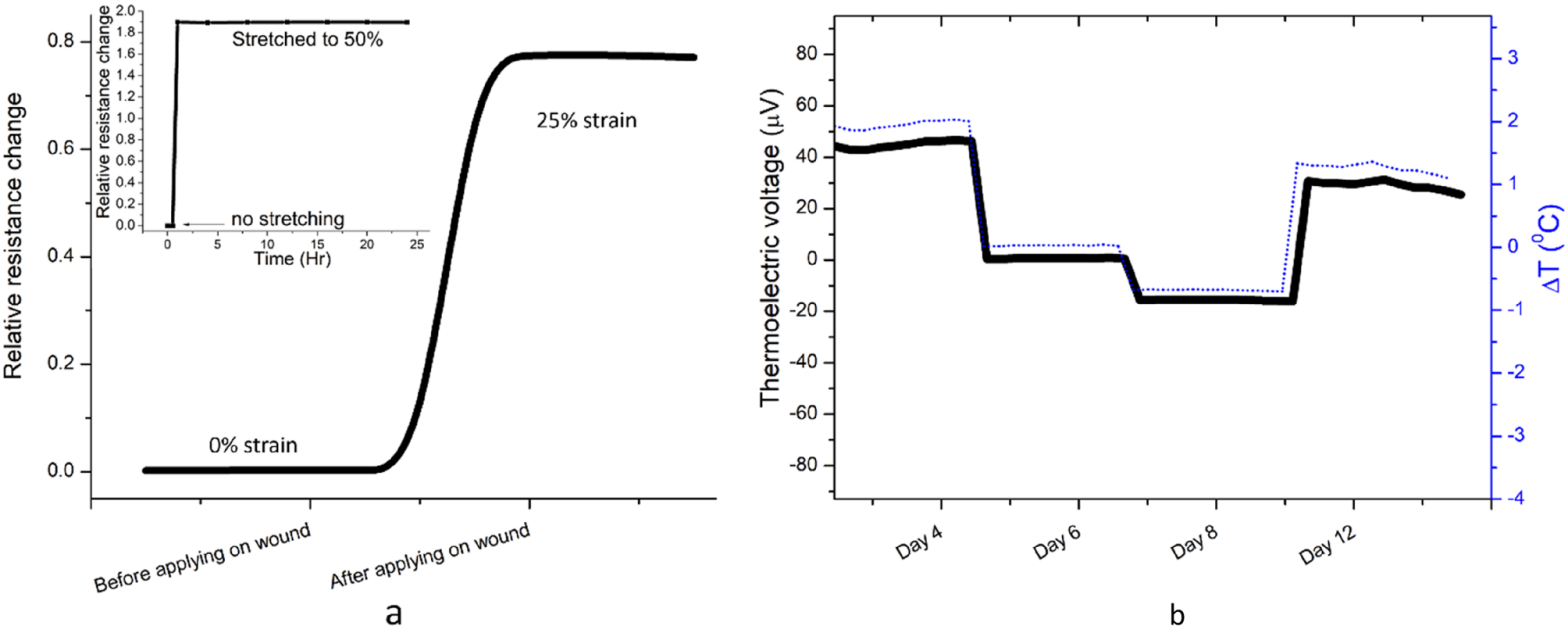
Strain induced resistance change upon applying the sensor on mice body (**a**), sensor resistance change is measured while it is elongated to 50% using a stretch bench and maintained it in the same position for 24 h (**a**, insets). Thermoelectric voltage generated during the test period of 4–12 days and corresponding ΔT is also shown in **b**.

As already mentioned in the methods and materials section, the mice experiments are carried out with sensors fabricated on Thermoplastic Polyurethane (TPU) which is more likely a onetime usable bandage. TPU foils are chosen as they are commonly applied in wound dressings because of their features like transparency, stretchability (the foil’s force -strain plot is shown in supplementary information S5.), breathability and biocompatibility^70,71^. The hard and soft polymeric segments of TPU are responsible for many of their interesting mechanical and physical properties. Moreover, the excellent printability of PEDOT:PSS on TPU and its structural integrity has been demonstrated in several stretchable applications^72,73^. Medical researchers recognized the significance of the modest distracting force on the wound strength from the experimental studies on multiple animals and they emphasized the necessity to quantify the stress on it^51^. The strain measurements are calculated from the relative resistance change and they show a GF of 4 for 25% stretching. This way one could appropriately set adequate tensile forces (pressure) at the wound site (Fig. 10a). To ensure the stability in the sensor readings, the sensor has been applied a strain value of 50% and is set to a state of continuous stretching for 24 h. Figure 10b (in the inset) shows that the resistance of the sensor remained constant over the entire stretching period which indicates that the strain sensor is capable of prolonged wound monitoring. In this study, the sensor was used to quantify the strain for sufficient tension at the wound site; however, difficulty in the extended placement of the sensor on the body of the mice subsequently after the sedation phase restricts further investigation in to the strain measurements. This investigation did not extend towards the study of the impact of mechanical forces on enhanced wound healing as it is well established in the medical literature. Instead, our work shows how to precisely set the right quantity of tension in the wound site with the help of this proposed strain sensor.

The proposed temperature sensor for mice wound measurements has a sensitivity of 24 μV/°C. The measurements are restricted to 4 to 5 min during which the sedation remains effective and a more prolonged sedation will lead to a significant drop of the mice body temperature. The relative temperature changes throughout the wound healing stages are shown in Fig. 10b. As it visually elucidates, the sensor shows an acceptable range of temperature difference during these measurements. On day 4, the wounds were slightly warmer with respect to the reference body temperature. By the 6th day, the temperature at the wound site became almost similar at both junctions. On the same day, after the measurements, the wounds were left open without any bandages. This is reflected in the readings of day 8, where the wounds were relatively colder as the wound closure was not completely occurred. Similar findings are also reported for wound temperature variations related to wounds with and without dressings^74^. Although the wounds were kept open in air, the readings of day 12 displayed a temperature higher than that on day 8 at the wound site. This is a clear indication of the partial or complete closure of the wounds by then (see also Fig. 9). This study on mice confirms the feasibility of using the studied sensor for in vivo measurements.

## Conclusion and future studies

This work introduced a printed wearable dual parameter sensor developed based on an intelligent approach for the simultaneous evaluation of strain and temperature stimuli for wound monitoring. The proposed sensor possessing wearable attributes like high stretchability and conformability is realised implementing the simple printing and coating techniques facilitating economical mass production. The stretchable temperature sensing part of the sensor exhibits excellent linearity, sensitivity and is consistent with different stretching levels. Moreover, the chemical treatment of the sensor further helped to enhance the sensitivity twice. Likewise, the strain sensing part of the device performs exceptionally well for applied strain levels between 2 and 70% with a resolution below 1%. The sensor exhibits a linear transfer function and impressive consistency with modest sensitivity. The addition of LiTFSI to the PEDOT:PSS based composition helped to effectively reduce the Young’s modulus of the functional layer which in turn enhanced the attributes of the strain sensor. Moreover, the wide range of sensing capabilities and the precision of the sensor projects it into exciting applications for wearable sensors.

The sensor effectivity has been verified on an animal model with an end goal of wound monitoring and the optimisation of the conditions for better healing. The sensor has been applied to monitor the wounds on the body of mice by tracking the temperature variations and by optimising the tensile forces at the wound site. This innovative sensor concept adds immense prospects and research interest towards smart wound monitoring. However, certain constraints arising from the complication in sensor placement in stretched mode on the mice dorsum due to their greater agitative nature and the rapid fall in body temperature due to prolonged sedation contribute to minor systematic errors in the measurements. Despite of these limitations, we successfully demonstrate the applicability of this innovative dual parameter sensor to measure different important wound parameters in an animal vivo model.

The future prospects of the demonstrated temperature-strain dual parameter sensor could be presumed within the realm of wearables where a smart wound dressing model is an example of this embodiment. A recent publication of S. Konishi & A. Hirata demonstrate a microfabricated temperature sensor integrated on the actuator of the micro fingers for sensation^75^. In the context of the present work, integration of a highly stretchable temperature -strain sensor into such micro fingers enables the precise governance of the finger movements. Such applications extend this work to versatile areas of robotics, where it needs accurate temperature sensation and precise measurement of the bending angles of the fingers. Thus, an innovative multifunctional device has been demonstrated employing printing electronics that enabled the fabrication on stretchable biocompatible substrates making them potential candidates in disposable wearable sensing that could perform in-vivo wound healing measurements, eliminating frequent bandage removal and permitting visual diagnosis.

## Supplementary Information

Supplementary Figures.
