# Supplementary material for: Stretchable printed device for the simultaneous sensing of temperature and strain validated in a mouse wound healing model
Source: Sci Rep. 2022 Jun 16;12:10138. doi: 10.1038/s41598-022-13834-6 (PMC9203561; doi:10.1038/s41598-022-13834-6)
Supplement: Supplementary file 1 — Supplementary Figures. [file 41598_2022_13834_MOESM1_ESM.docx]

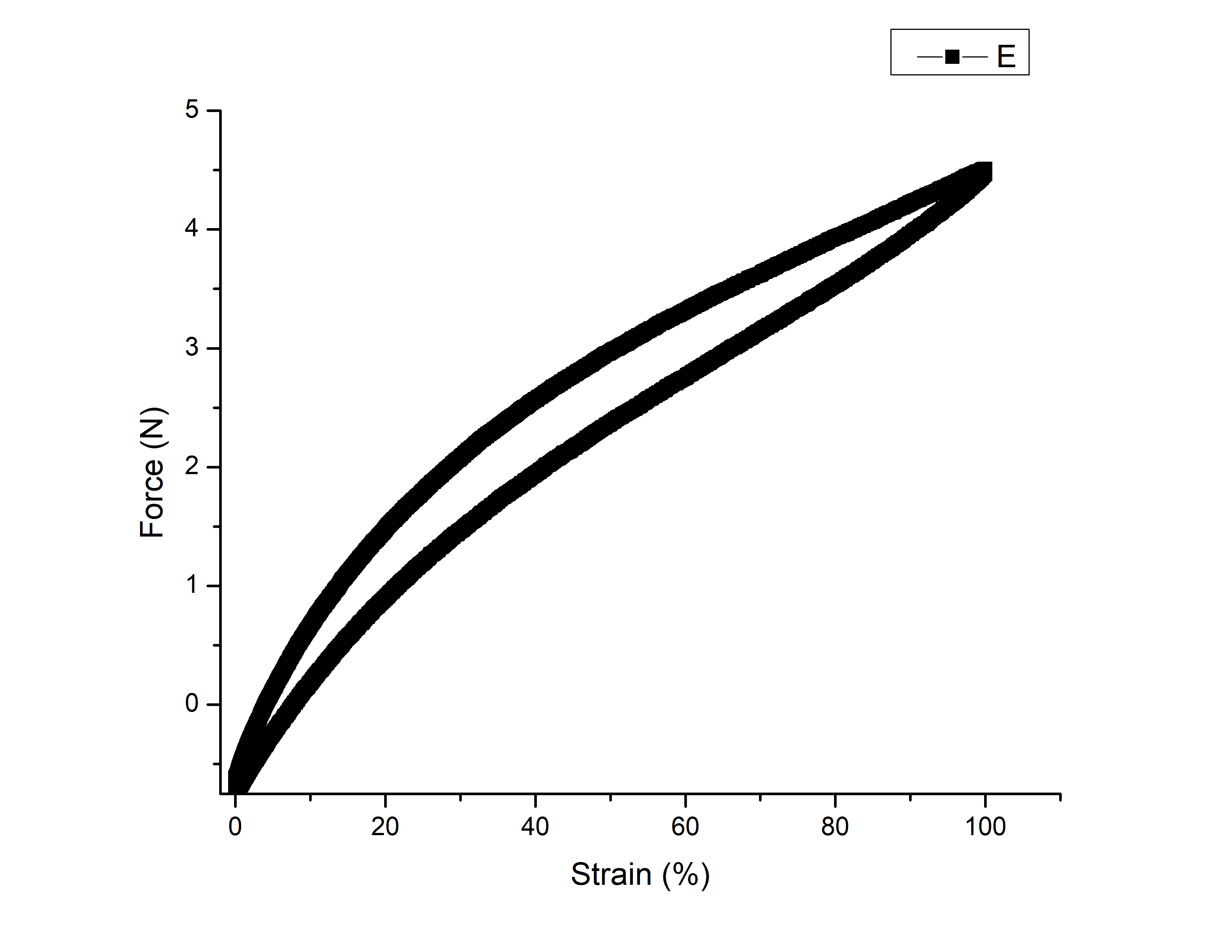
**Supplementary information**

Figure S1. The hysteresis curve between force and applied strain for the SEBS substrate.


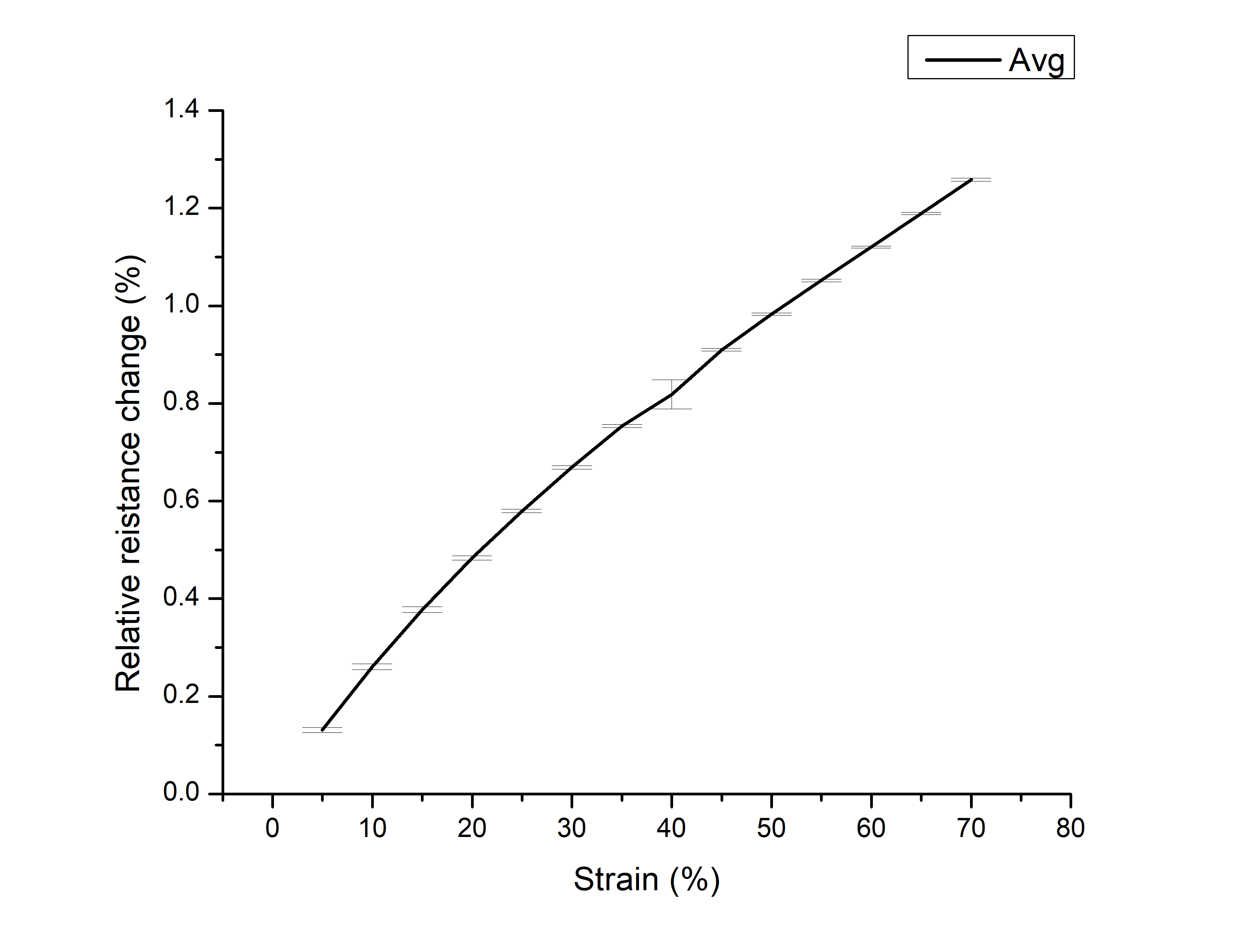


Figure S2. Plot between applied strain and relative resistance change with standard deviation.


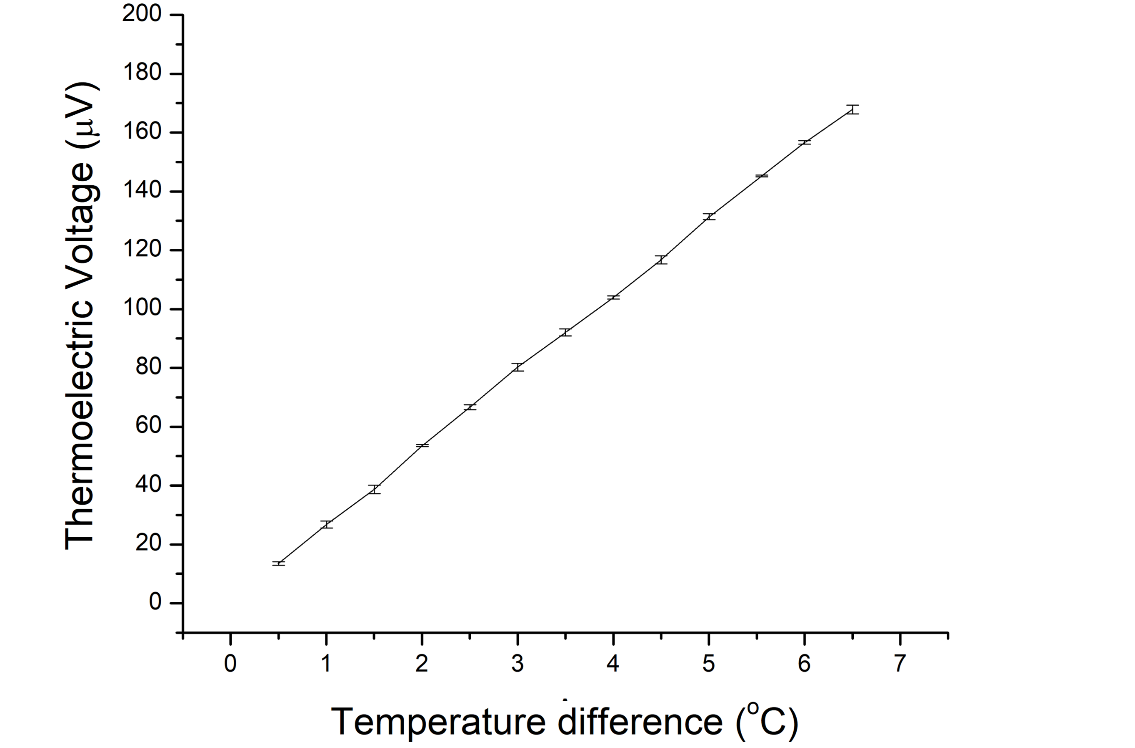


Figure S3. Plot between temperature difference and thermoelectric voltage measurements with standard deviation.


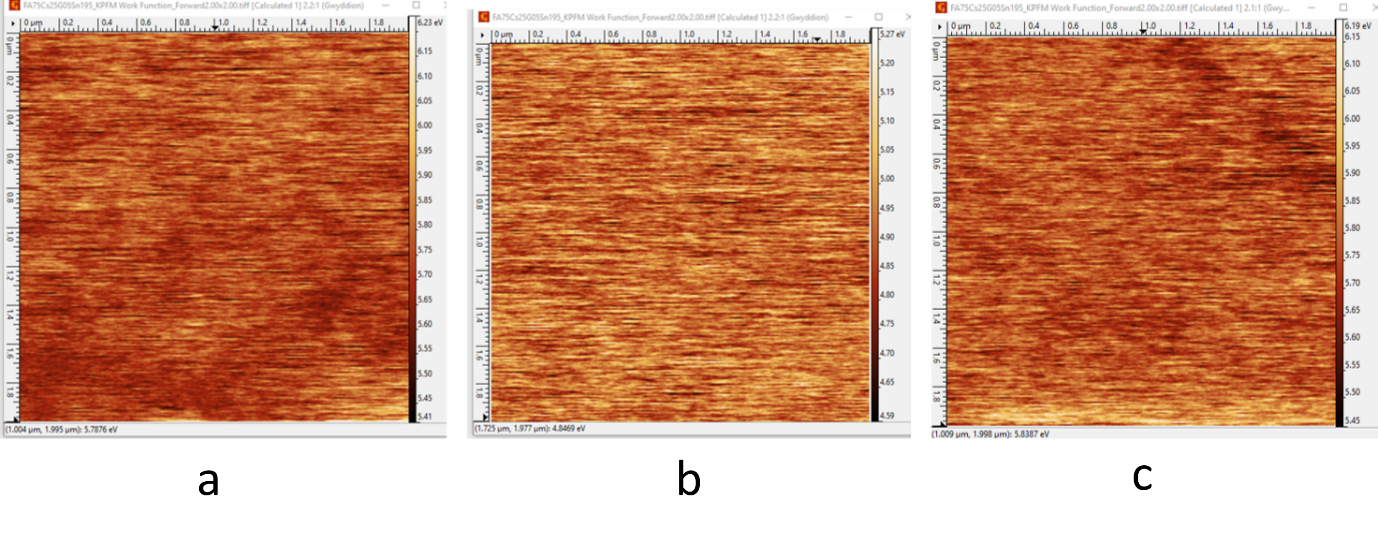


Figure S4. Surface potential KPFM imaging for PEDOT: PSS -LiTFSI film (a), for chemically treated (with Na_2_S_2_O_3_) PEDOT: PSS -LiTFSI film (b), PEDOT: PSS - LiTFSI film under stretch (c)


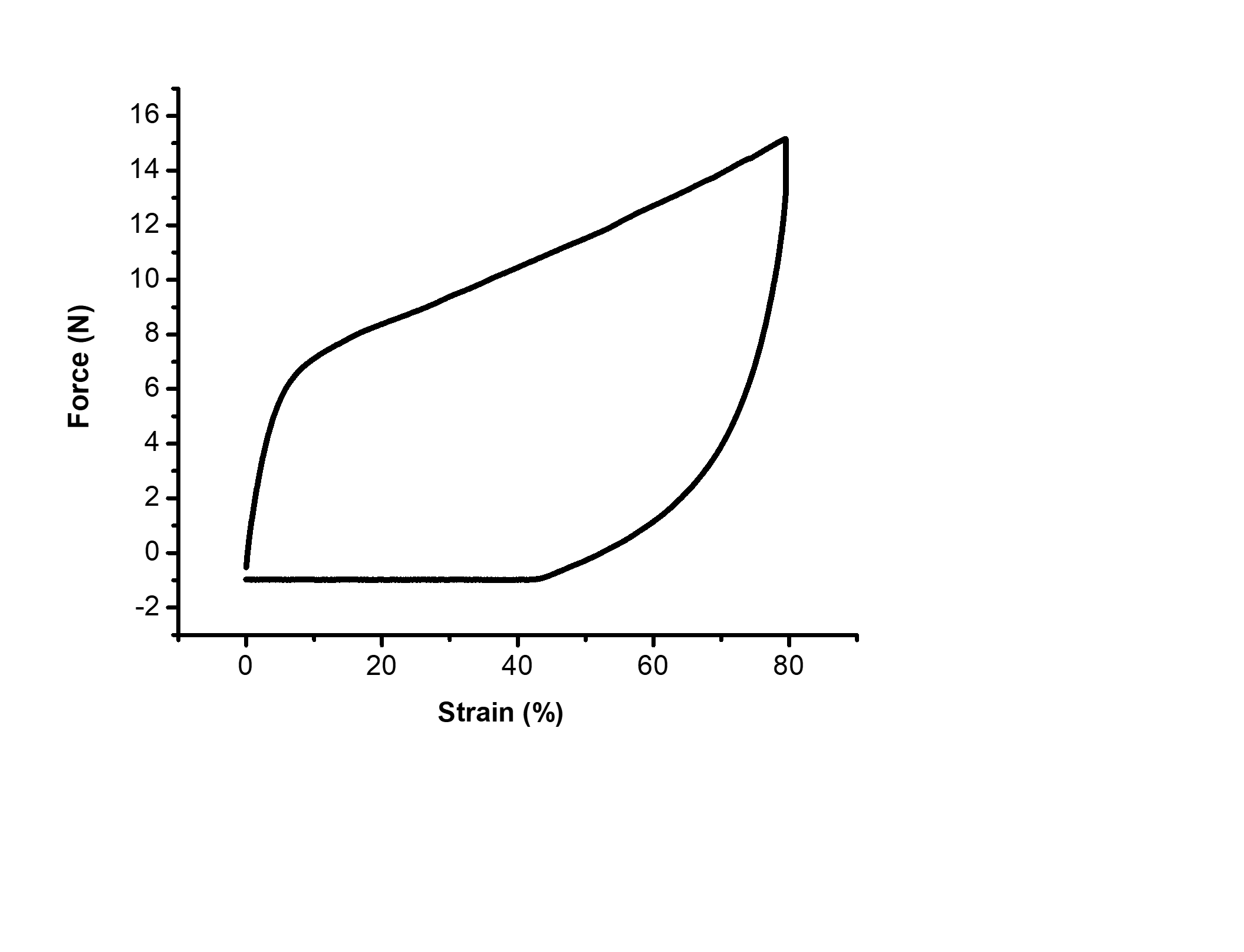


Figure S5. The force - strain plot for the SEBS substrate.
